# Supplementary material for: Application of hsp60 amplicon sequencing to characterize microbial communities associated with juvenile and adult Euprymna scolopes squid
Source: ISME Commun. 2025 Jun 13;5(1):ycaf085. doi: 10.1093/ismeco/ycaf085 (PMC12166977; doi:10.1093/ismeco/ycaf085)
Supplement: Smith-2024_Supplemental_Final_ycaf085 [file smith-2024_supplemental_final_ycaf085.pdf]

Supplemental Methods

Adult squid collection.

Adult *Euprymna scolopes* light organ cores were previously obtained by collecting wild-caught animals from Maunalua Bay with a dipnet, euthanized, ventrally dissected, and cores from each light organ lobe taken and stored at -80°C until DNA extraction.

Juvenile squid colonization.

Juvenile *Euprymna scolopes* were hatched into bacteria-free seawater and exposed to either a clonal inoculum of *V. fischeri* or to natural seawater. Animals were euthanized and whole animals were homogenized and stored at -80°C prior to DNA extraction.

Bacterial strains.

Bacterial isolates used in this study include *V. fischeri* ES114, *V. fischeri* MB13B1, *V. fischeri* MB13B2, *Rossellomorea aquimaris* TF-12, *Pseudoalteromonas luteoviolacea* HI1, and *Vibrio litoralis* DSM17657. Individual overnight cultures were grown at 28°C in Luria-Bertani salt (LBS)[1] media and diluted to 1.0 OD_{600}.

In mixed culture treatments, equal volumes of individual cultures were combined to a total volume of 1 mL. Clonal treatments are comprised of 1 mL of the respective normalized culture. Individual replicates of each culture treatment were pelleted using centrifugation and stored at -80°C until DNA extraction.

For juvenile squid colonization experiments, individual overnight cultures were diluted 100x in seawater tryptone (SWT)[1] medium broth and grown with shaking at 28°C until mid-exponential phase (0.5 OD_{600}). Bacteria were diluted to a concentration of ~5,000 cells/mL in 50 mL of filter-sterilized ocean water (FSOW) in which newly hatched juvenile squid were transferred. Incubations were performed at room temperature, between 21 and 24°C.

DNA extraction and *hsp60* amplicon sequencing.

DNA was extracted simultaneously from all samples using a Zymo Quick-DNA Fungal/Bacterial Miniprep kit (Zymo Research, Irvine, CA, USA) along with duplicate negative extraction controls (NECs). NECs consisted of sterile PBS (no sample) processed alongside samples through all experimental steps including DNA extraction and elution in DNA-free elution buffer, library preparation, sequencing, and subsequent bioinformatics analysis. NECs were included to control for any DNA contaminants that may be introduced during processing steps. 28 low-abundance ASVs detected in NEC controls were removed from experimental samples in post-processing as described in analysis documentation below. Amplicon sequencing of the *hsp60* gene was performed by UNC's High Throughput Sequencing Facility on a MiSeq System using primer sets H279/H280 and H1612/H1613 mixed at a 1:3 molar ratio as previously described [2-4]. See table S2 for amplicon read sequences processing summary.

Demultiplexed FastQ files were processed with DADA2 v1.10 [5]. The following parameters were used for sequence filtering and trimming: trimLeft=26, maxN=0, maxEE=2. The parameter trimLeft=26 was used to uniformly remove the primer sequence from demultiplexed forward reads. Due to amplicon length, only forward reads were analyzed in this study. Chimeric sequences were removed with the removeBimeraDenovo function of DADA2. Taxonomic classification of each ASV was performed with a naïve Bayesian classifier in QIIME2 [6] using p-confidence=0.6 and a custom *cpn60* reference database, available upon request (cpn60_classifier_v11.qza). This reference database comprises publicly available *cpn60* reference sequences [7] appended with the *hsp60* sequence and taxonomy of each bacterial isolate used in this study (*V. fischeri* ES114[8], *V. fischeri* MB13B1[9], *V. fischeri* MB13B2[9], *Rossellomorea aquimaris* TF-12[10], *Pseudoalteromonas luteoviolacea* HI1[11], and *Vibrio litoralis* DSM17657[12]). The identification of sequencing contaminants was assessed using

decontam v1.12 [13] based on frequency and input DNA concentration, as well as prevalence in negative extraction controls. Diversity analyses were performed with PhyloSeq v1.40.0 [14]. Relative abundance was assessed with the microViz v0.12.4 package [15]. See Escolopes_hsp60_Analysis for Rmd documentation of the bioinformatic analyses performed.

Data availability statement.

The amplicon sequences presented in this study are available via NCBI under BioProject PRJNA1136500.

Ethics statement.

The University of Hawaii Institutional and Animal Care and Use Committee (IACUC) is only allowed to review research using vertebrate animals. Author ER has a letter from the University veterinarian that states the use of cephalopods in the research conducted in this study would pass IACUC standards if they were allowed to formally review it.

Supplemental References.

1. Christensen, D.G., J. Tepavčević, and K.L. Visick, *Genetic manipulation of Vibrio fischeri.* Current protocols in microbiology, 2020. **59**(1): p. e115.
2. Hill, J.E., et al., *cpnDB: a chaperonin sequence database.* Genome research, 2004. **14**(8): p. 1669-1675.
3. Hill, J.E., J.R. Town, and S.M. Hemmingsen, *Improved template representation in cpn60 polymerase chain reaction (PCR) product libraries generated from complex templates by application of a specific mixture of PCR primers.* Environmental microbiology, 2006. **8**(4): p. 741-746.
4. Vancuren, S.J. and J.E. Hill, *Update on cpnDB: a reference database of chaperonin sequences.* Database, 2019. **2019**.
5. Callahan, B.J., et al., *DADA2: High-resolution sample inference from Illumina amplicon data.* Nature methods, 2016. **13**(7): p. 581-583.
6. Bolyen, E., et al., *Reproducible, interactive, scalable and extensible microbiome data science using QIIME 2.* Nature biotechnology, 2019. **37**(8): p. 852-857.
7. Ren, Q. and J.E. Hill, *Rapid and accurate taxonomic classification of cpn60 amplicon sequence variants.* ISME communications, 2023. **3**(1): p. 77.
8. Boettcher, K. and E. Ruby, *Depressed light emission by symbiotic Vibrio fischeri of the sepiolid squid Euprymna scolopes.* Journal of Bacteriology, 1990. **172**(7): p. 3701-3706.
9. Wollenberg, M.S. and E.G. Ruby, *Phylogeny and fitness of Vibrio fischeri from the light organs of Euprymna scolopes in two Oahu, Hawaii populations.* Isme j, 2012. **6**(2): p. 352-62.
10. Yoon, J.H., et al., *Bacillus marisflavi sp. nov. and Bacillus aquimaris sp. nov., isolated from sea water of a tidal flat of the Yellow Sea in Korea.* Int J Syst Evol Microbiol, 2003. **53**(Pt 5): p. 1297-1303.
11. Arnold, B.J., I.-T. Huang, and W.P. Hanage, *Horizontal gene transfer and adaptive evolution in bacteria.* Nature Reviews Microbiology, 2022. **20**(4): p. 206-218.
12. Nam, Y.D., et al., *Vibrio litoralis sp. nov., isolated from a Yellow Sea tidal flat in Korea.* Int J Syst Evol Microbiol, 2007. **57**(Pt 3): p. 562-565.
13. Davis, N.M., et al., *Simple statistical identification and removal of contaminant sequences in marker-gene and metagenomics data.* Microbiome, 2018. **6**: p. 1-14.
14. McMurdie, P.J. and S. Holmes, *phyloseq: an R package for reproducible interactive analysis and graphics of microbiome census data.* PloS one, 2013. **8**(4): p. e61217.
15. Barnett, D.J., I.C. Arts, and J. Penders, *microViz: an R package for microbiome data visualization and statistics.* Journal of Open Source Software, 2021. **6**(63): p. 3201.
